# Immunosuppressive network promotes immunosenescence associated with aging and chronic inflammatory conditions

**DOI:** 10.1007/s00109-021-02123-w

**Published:** 2021-08-25

**Authors:** Antero Salminen

**Affiliations:** Department of Neurology, Institute of Clinical Medicine, University of Eastern Finland, P.O. Box 1627, 70211 Kuopio, Finland

**Keywords:** Aging, Alzheimer’s, Cellular senescence, Immune tolerance, Immunosuppression, Kynurenine

## Abstract

The functional competence of the immune system gradually declines with aging, a process called immunosenescence. The age-related remodelling of the immune system affects both adaptive and innate immunity. In particular, a chronic low-grade inflammation, termed inflammaging, is associated with the aging process. Immunosenescence not only is present in inflammaging state, but it also occurs in several pathological conditions in conjunction with chronic inflammation. It is known that persistent inflammation stimulates a counteracting compensatory immunosuppression intended to protect host tissues. Inflammatory mediators enhance myelopoiesis and induce the generation of immature myeloid-derived suppressor cells (MDSC) which in mutual cooperation stimulates the immunosuppressive network. Immunosuppressive cells, especially MDSCs, regulatory T cells (Treg), and M2 macrophages produce immunosuppressive factors, e.g., TGF-β, IL-10, ROS, arginase-1 (ARG1), and indoleamine 2,3-dioxygenase (IDO), which suppress the functions of CD4/CD8T and B cells as well as macrophages, natural killer (NK) cells, and dendritic cells. The immunosuppressive armament (i) inhibits the development and proliferation of immune cells, (ii) decreases the cytotoxic activity of CD8T and NK cells, (iii) prevents antigen presentation and antibody production, and (iv) suppresses responsiveness to inflammatory mediators. These phenotypes are the hallmarks of immunosenescence. Immunosuppressive factors are able to control the chromatin landscape, and thus, it seems that the immunosenescence state is epigenetically regulated.

## Introduction

**Fig. 1:**
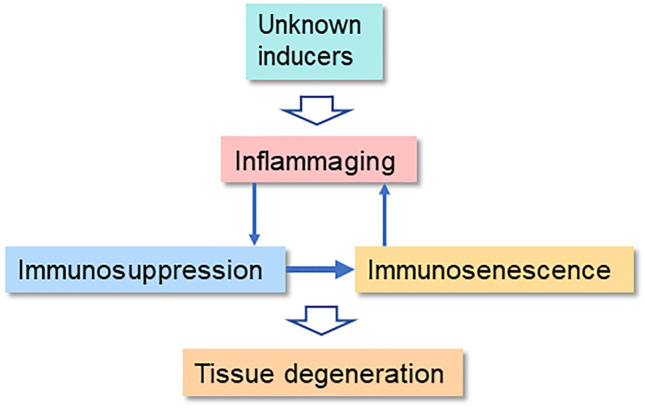
The feed-forward cycle between inflammaging, immunosuppression, and immunosenescence. Unknown inducers provoke an inflammaging state which stimulates a counteracting compensatory immunosuppression in an attempt to protect the host tissues. The activation of the immunosuppressive network (Fig. 2) induces an immunosenescence state in the effector immune cells. Subsequently, immunosenescence promotes inflammation and aggravates inflammaging in tissues. The chronic presence of both immunosuppression and immunosenescence augments tissue degeneration with aging and inflammatory conditions

**Fig. 2:**
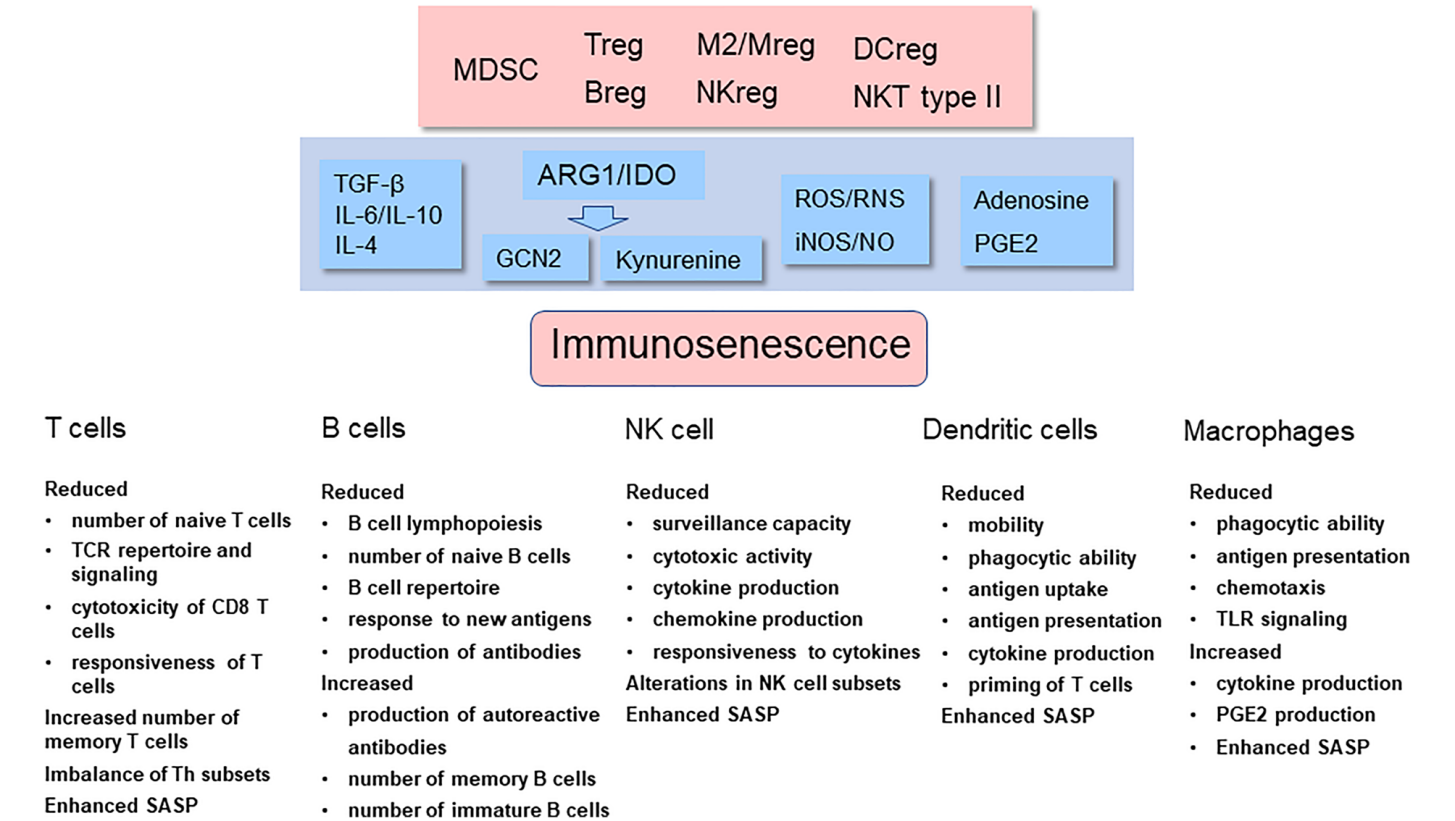
The figure depicts the generation of the immunosenescence phenotypes of different immune cells through the activation of immunosuppressive network. Immunosuppressive cells, e.g., MDSCs, Tregs, and M2 macrophages, secrete many factors which induce the immunosenescent phenotype of T and B cells as well as that of NK cells, dendritic cells, and macrophages. The molecular mechanisms have been described in the text. The functional properties of immunosenescent cells are listed below. Abbreviations: ARG1, arginase-1; Breg, regulatory B cells; DCreg, regulatory/tolerant dendritic cells; GCN2, general control nonderepressible 2; IL, interleukin; iNOS, inducible nitric oxide synthase; M2/Mreg, M2 macrophage (regulatory macrophage); MDSC, myeloid-derived suppressor cells; NKreg, regulatory natural killer cells; NKT type II, type II natural killer T cells; PGE2, prostaglandin E2; RNS, reactive nitrogen species; ROS, reactive oxygen species; SASP, senescence-associated secretory phenotype; TCR, T cell receptor; TGF-β, transforming growth factor-β; TLR, Toll-like receptor; Treg, regulatory T cells

The aging process is associated with a deterioration of the immune system, commonly referred to as immunosenescence. The age-related decline in the functions of immune cells involves both adaptive immunity, such as T and B cells, and innate immunity including macrophages as well as dendritic and natural killer (NK) cells [1, 2]. Concurrently, there exists a chronic low-grade inflammatory state, termed inflammaging (Fig. 1). At present, there is an on-going debate whether immunosenescence is a cause or a consequence of inflammaging. There is clear evidence that immunosenescence increases the risk for the growth of tumors and a persistent inflammatory state during infections, whereas it seems to improve the efficiency of transplantation [3–5]. Currently, the molecular basis of immunosenescence is unknown, although it is a hallmark of the aging process. Another characteristic of aging is the accumulation of senescent non-immune cells within tissues in both mice and humans [6]. Interestingly, not only inflammaging but also immunosenescence occurs in several pathological conditions associated with chronic inflammation (see below). It is known that persistent inflammation stimulates a counteracting compensatory immunosuppression intended to protect tissue homeostasis [7–9]. Immune cells are highly plastic cells which can display different phenotypes and even be converted into other immune cell types. These flexible processes are most probably under some form of epigenetic regulation (see below). Inflammatory mediators enhance myelopoiesis and trigger the generation of immature myeloid-derived suppressor cells (MDSC) which are able to enhance the immunosuppressive properties of other immune cells [10]. The immunosuppressive phenotypes are called regulatory ones including, e.g., regulatory T (Treg) and B (Breg) cells. Interestingly, immunosuppressive cells secrete different immunoregulatory factors which inhibit the acute inflammatory responses of effector cells and in that way promote their immunosenescence (Fig. 2). Here, I will examine the generation of immunosenescence, particularly the form encountered in T and NK cells, through the control of immunosuppressive network.

## Definition of immunosenescence and exhaustion of immune cells

The gradual decline of the immune system with aging, i.e., immunosenescence, is an evolutionarily conserved phenomenon. Immunosenescence represents the age-related remodelling of the immune system with aging [1, 2]. However, the definition of immunosenescence and its effects on human health with aging are currently under debate [11]. There are several reasons that immunosenescence seems to be an irreversible, context-dependent process, and it is not known whether the changes are beneficial or detrimental. In fact, the cells of the immune system can display some plasticity in their phenotypes. For instance, the T cell population can express the effector phenotypes (Teff) and immunosuppressive phenotypes, i.e., Treg cells. Accordingly, Tregs can provoke the changes in human T cells which are reminiscent of those found in immunosenescent T cells [12, 13] (Fig. 2). Moreover, human natural killer (NK) cells undergo a significant differentiation process with aging [14]. Given that the inflammatory microenvironment augments immunosenescence, the occurrence of immunosenescent cells increases in many age-related diseases [15, 16]. The presence of low-grade chronic inflammation most probably promotes immunosenescence with aging and thus enhances the inflammaging state through a feed-forward process [17]. Considering the plasticity of immune cells, it seems that there does not exist any specific immunosenescent phenotypes but instead a wide spectrum of immune cells expressing a set of markers of immune senescence. The characteristics of the immunosenescent phenotypes of different immune cells will be examined in detail below.

The age-related involution of the thymus has a profound effect on immunosenescence, especially on the senescence of mouse and human T cells. Studies on senescent T cells have revealed a state called T cell exhaustion which have many different properties compared to those of senescent T cells [18, 19]. Chronic infections, autoimmune diseases, and cancers, i.e., the chronic elevation of the antigenic load, induce the phenotype of exhausted T cells, in both CD4 and CD8 T cells. The exhaustion of Teff cells arrests cell proliferation and induces a hyporesponsive, anergic state which may safeguard against the development of autoimmune diseases. Exhausted T cells do not produce cytokines, whereas senescent T cells secrete an increased level of pro-inflammatory cytokines. Moreover, the specific hallmark of exhausted T cells is a significant increase in the expression of multiple inhibitory receptors, such as PD-1, CTLA-4, LAG-3, and TIGIT [19]. Wang et al. [20] exploited single-cell transcriptomics to assay the specific properties of exhausted T cells in the HIV-infected humans. They reported that exhausted CD8 T cells displayed a strong upregulation of killer cell lectin–like receptor subfamily G member 1 (KLRG1) in HIV patients. Interestingly, an antibody blockade therapy targeted at the KLRG1 protein significantly restored the function of CD8 T cells in HIV individuals. It is known that a therapy aimed at blocking the PD-1 protein also reinvigorated exhausted T cells in persistent virus infections and cancers [21]. There are studies indicating that human NK cells can display different dysfunctional states including anergy and exhaustion, in addition to the immunosenescent state [22]. Exhausted NK cells undergo an upregulation of PD-1 and KLRG1 receptors, whereas cytokine production is downregulated. In contrast, senescent NK cells show an increased secretion of pro-inflammatory cytokines (see below). An interesting difference between the exhaustion and senescence of NK cells is the observation that the exhausted state of NK cells can be reversed by receptor blockade therapy, but the senescence state is more stable. Currently, the signaling mechanisms controlling the senescence and exhaustion of both T and NK cells are under scrutiny because of their important role in chronic inflammatory diseases.

## Hallmarks of immunosenescence

### Functional immune hallmarks

The characteristics of immunosenescence have normally been assessed through the changes appearing in the surface markers and functional properties of different immune cell populations. There is a plethora of articles which have described in detail the typical changes in the phenotypes of senescent immune cells in mice and humans [17, 23–25]. The common functional alterations appearing in the immunosenescent state of different immune cells have been collected to Fig. 2. In T cells, there is a gradual decline in the number of naïve CD4^+^ and CD8^+^ T cells (CD45RA^+^), whereas those of the memory phenotype (CD45RO^+^) progressively increase during the aging process [23, 26]. The decrease in the number of CD4^+^ and CD8^+^ T cells is attributable to the reduced clonal expansion of T cells in the bone marrow (BM) and the involuting thymus during the aging process. In the BM, there exists an age-related myeloid-biased dominance of human hematopoiesis, whereas the production of lymphopoietic progenitors is downregulated with aging [27]. Moreover, the responsiveness of T cells to certain cytokines declines with aging as does the cytotoxic activity of CD8^+^ T cells (Fig. 2). There is convincing evidence that immunosenescence is associated with disturbances in the function of the T cell receptor (TCR) and its co-receptors, especially that of the stimulatory CD28 receptor. Particularly, a loss of CD28 receptors is a hallmark of senescent T cells [25]. The clear decrease in the diversity of the human TCR repertoire with aging in both naïve CD4^+^ and CD8^+^ T cell populations impairs the recognition of antigens and reduces the activation of T cells [28]. In addition to T cells, the number of B cells also declines with aging (Fig. 2). Kennedy and Knight [29] revealed that MDSCs inhibited the generation of B cells in mouse BM cultures through the secretion of soluble factors. The soluble factors could be anti-inflammatory cytokines since TGF-β is a potent inhibitor of B cell proliferation [30]. There are also age-related changes in the circulating B cell subsets, i.e., the number of human naïve B cells and the antigen-experienced memory (CD27^+^) B cells are reduced with aging, whereas the presence of the exhausted memory (CD27^−^) B cells increases [31]. Aging also affects the B cell repertoire and antibody production, e.g., the production of autoreactive antibodies is augmented with aging (Fig. 2). Frasca et al. [31] have reviewed in detail the changes present in the phenotypes and functions of senescent B cells in mice and humans.

NK cells are important cytotoxic cells which undertake immune surveillance and clearance of stressed cells in cooperation with cytotoxic CD8^+^ T cells. Considering the aging process, it does appear that senescent cells can be recognized and removed by NK cells [32]. It seems that the accumulation of senescent cells with aging is attributable to defects in the surveillance potential of NK cells [33]. There is substantial evidence that aging affects the diversity of NK cell subsets and thus the functional properties of mouse and human NK cells [14, 34]. The common alterations in the profiles of surface receptors appearing in NK cells with aging have been compiled in several reviews [14, 34, 35]. In brief, the number of CD56^bright^ NK cells declines in circulation with aging as well as the production of cytokines and chemokine. Moreover, the percentage of CD56^dim^CD57^pos^ NK cells increases with aging, whereas that of CD56^dim^CD57^neg^ cells is clearly downregulated. Interestingly, the CD57^pos^ phenotype is a marker for the senescence state in human CD8^+^ T cells, whereas in NK cells, it represents terminal differentiation [36]. The frequencies of CD57^pos^ cells are increased in the blood and tissues in chronic inflammatory conditions, such as chronic infections and cancers. Almeida-Oliveira et al. [35] demonstrated that the percentages of human natural cytotoxicity triggering receptors 1 (NCR1 or NKp46) and 3 (NCR3 or NKp30) in both CD56^brigh^ and CD56^dim^ NK subsets were significantly reduced in elderly people. Moreover, Hazeldine et al. [37] reported that the release of perforin from human NK cells and its binding to target cells at the immunological synapse were clearly reduced with aging, thus decreasing the cytolytic activity of NK cells. These age-related alterations might explain the lower cytotoxicity of NK cells in aged individuals. Rajagopalan and Long [38] demonstrated that the sustained activation of KIR2DL4 (CD158d) receptor induced the senescence phenotype of human NK cells. They also reported that TRAF6/TAK1 signaling was able to activate the endosomal KIR2DL4 receptor and thus provoked the senescence of human NK cells. The TRAF/TAK1 signaling axis has several connections to TGF-β and NF-κB signaling. Hazeldine and Lord [34] have examined in detail many alterations in NK cells associated with the aging process.

The age-related changes in the myeloid cells, such as monocytes, neutrophils, macrophages, and dendritic cells, representing innate immunity are far less consistent than those of T, B, and NK cells. Their properties are more dependent on changes in the tissue microenvironment, e.g., the phase and intensity of inflammation and the presence of immunosuppressive cells. Since the major function of dendritic cells (DC) is antigen presentation to T and B cells, they are able to control the efficiency of adaptive immunity. Furthermore, macrophages possess a wide context-dependent plasticity, e.g., the M1/M2 polarization is not only dependent on the inflammatory state, but changes in the extracellular matrix can also affect their activity [39]. The number of DCs seems to be stable in tissues with aging, although the amount of Langerhans cells decreases with aging in human skin [40]. However, certain key functions of DCs are impaired with aging, e.g., mobility and antigen uptake are decreased, and also antigen processing and presentation to T cells are downregulated [41, 42] (Fig. 2). The priming of T cells might also be impaired, since there exists a significant decline in the secretion of cytokines induced by the activation of Toll-like receptors (TLR) in human myeloid and plasmacytoid DCs [43]. There is clear evidence that the macrophages from aged humans display many functional deficiencies [44, 45]. For instance, phagocytic capacity and the activity of antigen presentation are significantly reduced in senescent macrophages. Their responses to inflammatory insults, e.g., to LPS exposure and infections, are considerably downregulated, impairing the resolution of inflammatory state. However, it is known that the age-related effects on macrophages are dependent on the type of insult and the tissue location of the macrophages [46].

### Similar characteristics of senescence in immune and non-immune cells

The common hallmarks of cellular senescence are very well characterized in non-immune cells, whereas the cellular properties of senescent immune cells have been infrequently reported. Surprisingly, it has been demonstrated that there are major similarities in the common phenotypes between senescent immune and non-immune cells [6, 47, 48] (Table 1). The arrest of cell proliferation caused by an increase in the expression of cell cycle inhibitors, such as p16INK4a, p21WAF1, and p53, is a typical alteration encountered both in senescent immune and non-immune cells of mice and humans. For instance, these changes are present in senescent T cells [49–51], B cells [31], NK cells [52], and macrophages [53–55]. Cell cycle arrest is commonly associated with the increased expression of senescence-associated β-galactosidase (SA-β-gal) and heterochromatin foci (SAHF) [52–54, 56, 57] as well as the shortening of telomeres [58–60]. In particular, telomere shortening occurs in the replicative senescence of T cells. All these age-related changes are also common biomarkers of cellular senescence in non-immune cells of mice and humans [6] (Table 1).

**Table 1:** Comparison of common characteristics shared by cellular senescence and immunosenescence

**Parameter**	Cellular senescence	Immunosenescence	Immune cell type	Reference
SA-β-Gal	Up	Up	T, NK, M	[52–54, 56]
SAHF	Up	Up	T, NK	[52, 57]
Telomere shortening	Up	Up	T, B, NK, M	[58–60]
p16/INK4a	Up	Up	T, B, M	[49, 53, 54]
p21/WAF1[	Up	Up	T, NK, M	[50, 52, 55]
p53	Up	Up	T, M	[51, 55]
Autophagy	Impaired	Impaired	T, M	[56, 61–63]
Mitochondrial disturbances	Increased	Increased	T, B	[60, 63, 66]
Oxidative stress	Increased	Increased	T, B, M	[59, 66–68]
ER stress	Increased	Increased	T, M	[64, 65]
SASP	Enhanced	Enhanced	T, B, M	[55, 68, 70, 71]
Apoptosis	Resistant	Resistant?	T, B	[75–77]

Immune cells: *T* T cells, *B* B cells, *NK* natural killer cells, *M* macrophages

Diverse cellular stresses are associated with cellular senescence, both in immune and non-immune cells. There are significant alterations in the functions of autophagy, endoplasmic reticulum (ER), and mitochondria leading to cellular senescence displaying oxidative stress and disturbances in energy metabolism and protein homeostasis, although senescent cells remain metabolically active. There is robust evidence that the functions of autophagy [56, 61–63], ER [64, 65], and mitochondria [60, 63, 66] are disturbed in senescent mouse and human immune cells (Table 1). Currently, it is not known which of these three organelles is driving the senescence process, in either non-immune or immune cells. Several studies have revealed that autophagic degradation is reduced in senescent CD4^+^ T cells and macrophages which impair the clearance of mitochondria and other organelles, thus provoking disturbances in their function [61, 63]. Bektas et al. [63] demonstrated that an age-related decline in autophagic degradation in human CD4^+^ T cells induced the accumulation of autophagosomes and increased the number of non-functional mitochondria. Stranks et al. [61] reported that the knockout of the mouse *Atg7* gene, an essential autophagy gene, induced several typical properties occurring in immunosenescent macrophages. For instance, impaired autophagy in mouse macrophages reduced mitochondrial respiration and increased glycolysis, whereas it increased the secretion of inflammatory factors. Hurst et al. [64] reported that an increased ER stress induced mitochondrial exhaustion in mouse and human T cells. Interestingly, they reported that an increase in the generation of mitochondrial ROS compounds provoked a mitochondrial collapse in T cells. An increased production of mitochondrial ROS can also trigger telomere attrition in aged human CD8^+^ T cells [60]. Vida et al. [67] demonstrated that oxidative stress increased with aging in conjunction with a decrease of antioxidant defence in murine peritoneal leukocytes and especially in macrophages. The increases in the levels of ROS compounds and oxidized glutathione (GSSG) with an accumulation of lipofuscin impaired the functioning of senescent mouse macrophages. Interestingly, they observed that the macrophages from the long-lived mice preserved better their redox state and immune functions indicating that immunosenescence might enhance the oxi-inflamm-aging process. Accordingly, Garrido et al. [68] revealed that peritoneal macrophages and leukocytes sampled from the mice of two models of premature aging displayed decreased antioxidant levels accompanied by an increased quantity of oxidants and pro-inflammatory cytokines. Recently, Martinez de Toda et al. [69] screened a wide array of mouse immune cell parameters to determine which of them could be used as markers for the rate of the aging process. They reported that specific parameters were determinants of longevity in the adult age, e.g., lymphocyte chemotaxis and proliferation capacities as well as macrophage chemotaxis and phagocytosis activity. Furthermore, some other parameters predicted extreme longevity in very old age, such as the activity of NK cells and the levels of IL-6 and IL-1β cytokines. These results indicate that certain functional activities and inflammatory parameters of immune cells can be utilized as the prognostic tools for the prediction of human lifespan.

There are several studies indicating that the phenotype of senescent immune cells displays the characteristics of the senescence-associated secretory phenotype (SASP), a typical pro-inflammatory phenotype of senescent non-immune cells. The SASP response has been detected in T cells [25, 68, 70], B cells [71], and macrophages [55]. The pro-inflammatory SASP components include colony-stimulating factors (CSF), interleukins such as IL-6, IL-1β, IL-8, and IL-10, and several chemokines. It seems that the pro-inflammatory SASP properties of immune cells, especially those of macrophages, have a key role in the maintenance of low-grade chronic inflammaging condition in tissues. NF-κB signaling is the major inducer of the SASP state both in senescent immune and non-immune cells [55, 72]. The NF-κB system also is a potent regulator of apoptotic cell death, either inhibiting or enhancing apoptosis [73]. Cellular senescence of non-immune cells has been associated with extensive resistance to apoptotic cell death [74] (Table 1). Considering immunosenescence, the situation seems to be more complex, and apoptosis is probably a context-dependent process, i.e., immunosenescence either increases resistance to apoptosis or enhances apoptotic cell death. For instance, Spaulding et al. [75] demonstrated that the antigen-induced replicative senescence of human CD8^+^ T cells displayed a robust resistance to apoptosis. In contrast, Dennett et al. [76] reported that the age-related decline in the expression of CD25 and CD28 receptors in human T cells increased their susceptibility to the Fas (CD95)-mediated apoptosis. Chong et al. [77] reported that the number of apoptosis resistant CD27^−^ B cells was increased in aged humans. Currently, virtually nothing is known about the role of other regulated cell death processes, e.g., pyroptosis and immunogenic cell death, in the decline of immune system with aging.

## Immunosenescence is associated not only with aging but also with chronic inflammatory conditions

There is a debate whether immunosenescence is a cause or a consequence of a low-grade inflammation with aging [17]. It seems very plausible that immunosenescence is a consequence of inflammaging, since diverse inflammatory conditions, unrelated to the aging process, display extensive immunosenescent state of immune cells, e.g., many infections and autoimmune diseases [4, 26, 78]. For instance, rheumatoid arthritis induces premature T cell senescence displaying a deficiency of CD28 receptor and enhanced pro-inflammatory SASP properties [78, 79]. Immunosenescence is clearly increased in several age-related diseases, e.g., cardiovascular and neurodegenerative diseases [15, 16]. In fact, it seems probable that it is chronic inflammation rather than the aging process which augments immunosenescence. Cytomegalovirus infection can also enhance immunosenescence with aging. Recently, it has been reported that the infection by SARS-CoV2 virus induced an extensive immunosenescence response in older patients [80] which might be explained by the enhanced SASP activity of immune cells in aged individuals (see above). There is also convincing evidence that obesity accelerates immunosenescence in murine adipose tissues [81]. Evidently, it is related to chronic inflammation in adipose tissues rather than simply to the aging process. Moreover, neuropsychiatric disorders, e.g., major depression and bipolar disorder, are associated with chronic inflammation and immunosenescence [82, 83].

Immunosenescence has also been associated with tumorigenesis [5] and transplantation [84]. There is a debate whether immunosenescence in tumor sites might be attributed either to an age-related immune deficiency or to the chronic inflammatory microenvironment. Interestingly, in tumors, there is also an accumulation of senescent non-immune cells which are able to suppress tumor growth, but on the other hand, they can also enhance tumorigenesis through the proinflammatory SASP response [85]. When considering immunosenescence, T cells display the characteristics of senescence and exhaustion in the tumor microenvironment [19]. Moreover, Sanchez-Correa et al. [86] reported that human NK cells possessed features of immunosenescence in acute myeloid leukemia impairing immunosurveillance. It is known that the number of immunosuppressive regulatory cells, e.g., MDSCs, Tregs, and tumor-associated macrophages (TAM) robustly increases in tumor sites [87]. Given that regulatory immune cells are potent suppressors of the effector subsets of immune cells, this indicates that the activation of immunosuppressive network most probably induces immunosenescence in the tumor microenvironment. In addition, several studies have revealed that immunosuppressive cells, such as MDSCs, Bregs, regulatory macrophages (M2 subsets), and tolerogenic dendritic cells control transplantation tolerance in mice and humans [88]. This tolerance is most probably associated with the immunosenescence induced by the accumulation of immunosuppressive cells around the transplants. Recently, Sacchi et al. [89] reported that inflammation induced the expansion of a polymorphonuclear MDSC population in the blood of COVID-19 patients. They also demonstrated that MDSCs suppressed the specific responses of T cells to the SARS-CoV-2 virus-induced infection, e.g., inhibiting the release of IFN-γ cytokines. We have recently described that the age-related senescence of immune cells shows very similar phenotypes as those induced by immunosuppressive MDSCs [24]. As a whole, it seems that immunosenescence is caused by immunosuppressive cells as a reaction to the chronic inflammatory state (see below).

## Activation of immunosuppressive network promotes immunosenescence

### Immunosuppressive network

The immune system possesses extensive plasticity to respond and adapt to both systemic and local microenvironmental insults. There is convincing evidence that acute inflammatory responses evoke compensatory anti-inflammatory/immunosuppressive responses to induce a resolution phase both at the systemic level and in tissue microenvironments. Autoimmune diseases, pathogen-induced sepsis, and traumatic injuries are known to induce a systemic inflammatory response syndrome (SIRS) which is opposed by a compensatory anti-inflammatory syndrome (CARS) [7, 90]. In addition, chronic inflammatory states within tissues also provoke many immunosuppressive responses intended to counteract the harmful effects of persistent inflammation [8, 9]. The persistent presence of an immunosuppressive state can be detrimental, for instance, the chronic inflammation existing in tumor microenvironments provokes immunosuppression which allows tumor cells to escape immune surveillance [87]. The immunosuppressive network involves the regulatory Tregs, Bregs, NKregs, and DCregs, as well as M2 subsets of macrophages (Mregs) and type II NKT cells. The immature MDSCs, both monocytic and polymorphonuclear subpopulations (M-MDSC/PMN-MDSC), are also included in the immunosuppressive network [9, 91, 92] (Fig. 2). The close cooperation between the members of this immunosuppressive network is a characteristic property in the function of the network. For instance, the network’s members can potentiate each other’s immune suppressive activities and even enhance the differentiation of immunosuppressive cells [9, 91, 93]. The cells of host tissues are also able to educate immune cells to adopt immunosuppressive phenotypes [94]. Changes in the components of the extracellular matrix can also control the properties of immune cells in inflammatory sites [95]. These observations indicate that the plasticity of mouse and human immune cells allows the immune system to counteract inflammatory insults and thus maintain tissue homeostasis.

The cells of the immunosuppressive network possess powerful tools to suppress the functions of immune cells. The armament inhibits the functions of effector cells through (i) the secretion of anti-inflammatory cytokines, such as TGF-β, IL-4, IL-10, and IL-18, (ii) the release of reactive oxygen and nitrogen species (ROS/RNS), (iii) the generation of adenosine and prostaglandin E2 (PGE2) which are immunosuppressive factors, and (iv) increases in the expression and secretion of amino acid-catabolizing enzymes, i.e., arginase 1 (ARG1) and indoleamine 2,3-dioxygenase (IDO) [30, 96–98] (Fig. 2). Anti-inflammatory cytokines not only are immune mediators between immunosuppressive cells, but they also suppress the functions of pro-inflammatory immune cells and induce their immunosenescence. For instance, TGF-β exposure upregulates the expression of the cyclin-dependent kinase inhibitors thus arresting the proliferation of T cells as well as it inhibits the activation of T cells, disturbs the differentiation of Th cells, and inhibits the cytotoxicity of CD8 T cells [30]. TGF-β signaling also reduces the proliferation of B cells, inhibits antibody production, and induces apoptosis of immature and resting B cells. In human NK cells, TGF-β suppresses their cytolytic activity and the production of cytokines [30, 99]. Moreover, TGF-β exposure reduces the phagocytic activity and antigen presentation of macrophages [30]. All these phenotypes are reminiscent of those present in immunosenescent phenotypes of immune cells (Fig. 2). It is known that TGF-β can also arrest the cell cycle and induce cellular senescence of non-immune cells [100]. ROS/RNS compounds secreted by immunosuppressive cells inhibit the TCR signaling of mouse T cells as well as suppressing the cytotoxic activity, cytokine production, and signal transduction of rat NK cells [101, 102]. ROS/RNS compounds are also known to induce cellular senescence of non-immune cells [103]. Moreover, immunosuppressive cells, e.g., inducible Tregs, generate adenosine and prostaglandin E2 (PGE2) which are potent suppressors of effector T cells and NK cells [97, 104] (Fig. 2). Adenosine and PGE2 inhibit the functions of T cells and NK cells by stimulating cyclic AMP signaling. The robust increase in the production of PGE2 is associated with many human age-related inflammatory diseases, such as atherosclerosis [105].

The age-related decline in the function of immune system was discovered over 40 years ago [106–108]. Interestingly, these early studies indicated that immunosenescence was induced by the increased activity of immune suppressor cells [107, 108]. In their seminal study, Roder et al. [107] demonstrated that mouse spleen and bone marrow contained immune cells which were able to suppress the immune responses induced by anti-sheep erythrocytes (anti-SRBC). Interestingly, they revealed that the immunosuppressive response was robustly increased with aging and preceded or paralleled the age-related immunosenescence. The immunosenescence was mediated through the soluble factors from suppressor cells. In addition, Singhal et al. [108] reported that mouse spleen and bone marrow contained suppressive B-type cells which were able to inhibit the activation and differentiation of T and B lymphocytes through their secreted mediators. The activity of suppressor B cells was significantly increased with aging, and the cells were able to induce the age-related immunosenescence. After these ground-breaking observations, it took 30 years before many studies revealed that there was a significant age-related increase in the number of Tregs in the circulation of both humans [109, 110] and mice [111]. Accordingly, the number of MDSCs increased with aging in the blood of humans [112] and mice [113]. Currently, there are only a few studies which have focused on the alterations occurring in immunosuppressive cells within tissues during the aging process. For instance, with aging, there was an increased presence of immunosuppressive M2 macrophages in the mouse bone marrow, spleen, lungs, and skeletal muscles [114, 115]. Accordingly, it is known that the level of Tregs was upregulated with aging in mouse skin [116] and adipose tissues [117]. Ruhland et al. [118] demonstrated that the skin of elderly humans contained not only an increased number of senescent, INK4a-positive cells but that there was also a robust upregulation of immunosuppressive MDSCs. They also revealed that the experimentally induced senescence of stromal cells in the mouse skin provoked local inflammation and subsequently enhanced the expansion of MDSCs and Tregs. These studies clearly indicate that chronic inflammation can induce a counteracting activation of immunosuppressive network within tissues.

### Immature MDSCs are enhancers of immunosenescence

MDSCs are a heterogeneous population of immature myeloid cells which originate from the common myeloid progenitor cells in the bone marrow [10]. Many pathological processes disturb myelopoiesis, increasing the generation of immature myeloid cells (IMC) which can become differentiated into MDSCs. Inflammatory mediators, such as colony stimulating factors (CSF) and many chemokines, impair the maturation of MDSCs into macrophages, granulocytes, or dendritic cells. There is significant plasticity in the production of MDSCs and subsequently in their differentiation into the other myeloid cells [10, 119]. Basically, there are two types of MDSCs, i.e., M-MDSC and PMN-MDSC populations, which possess impressive immunosuppressive properties against different sets of immune cells. For instance, MDSCs are the potent inhibitors of the proliferation of T cells; this effect is mediated through the secretion of IL-6, IL10, and TGF-β cytokines as well as ROS and RNS compounds [120]. Nagaraj et al. [102] revealed that mouse MDSCs robustly released ROS compounds and nitric oxide (NO) forming peroxynitrite which nitrated the tyrosine residues of the TCR receptor. This process impaired the recognition of antigens, inhibited the signaling by TCRs inducing T cell tolerance which is a typical characteristic of immunosenescent T cells (Fig. 2). Kennedy and Knight [29] reported that mouse MDSCs inhibited the lymphopoiesis of B cells in bone marrow cultures. MDSCs also reduced the proliferation, homing, and antibody production of mature human B cells [121]. The suppression of B cells was mediated through the secretion of TGF-β, IL-10, and PGE2 by MDSCs. In contrast, MDSCs stimulated the expansion and activity of immunosuppressive Tregs [122] and Bregs [123]. This indicates that MDSCs have opposite effects on T and B cell populations in inflammatory conditions; they can either augment the immunosuppressive properties of Tregs and Bregs or suppress the immune activities of effector T and B cells evoking immunosenescent phenotypes.

It is known that MDSCs can also induce clear changes in the phenotypes of DCs, NK cells, and macrophages which resemble those present in the immunosenescent state [24] (Fig. 2). For instance, Poschke et al. [124] demonstrated that human M-MDSCs impaired the maturation of DCs, disturbed their ability to take up antigens, and inhibited the IFNγ production by human T cells. Hu et al. [125] also observed that mouse MDSCs actively secreted IL-10 cytokines which downregulated IL-12 production from DCs, thus suppressing their capacity to stimulate the functions of T cells. MDSCs are also potent suppressors of the cytotoxic functions of NK cells. For instance, Hoecht et al. [126] reported that the M-MDSCs, isolated from human hepatocellular carcinoma, suppressed the cytotoxicity of autologous NK cells and inhibited the secretion of cytokines. The suppression of NK cells disturbed the defense not only against tumors but also against viral infections [127]. Moreover, MDSCs are potent inhibitors of the immune surveillance performed by NK and CD8 T cells (see below). In inflammatory conditions, there exists a close interaction between MDSCs and tissue macrophages [128]. For instance, Nguyen et al. [129] demonstrated that during an infection in the mouse peritoneum, IL-10 induced the differentiation of blood monocytes into the MHCII^low^ macrophages possessing an elevated capacity to phagocytose apoptotic cells, whereas they were unable to present antigens to T cells. Given the abundant plasticity of tissue macrophages, Stout and Suttles [39] suggested that the immunosenescence of macrophages would be controlled by the changes in the extracellular matrix and immune cells present in aged tissues.

### Tregs induce T cell senescence

As discussed above, there is an opposite effect in the subtypes of T cells with aging, i.e., the number of naïve T cells declines extensively, whereas that of Tregs increases. There are several studies indicating that the members of the immunosuppressive network can convert T cells into immunosuppressive Tregs [91, 122]. The conversion of naïve and efferent T cells into the regulatory phenotype of T cells (Tregs) can be evoked by IL-10 and TGF-β cytokines which increase the expression of *FoxP3*, a master gene of Tregs. Conversely, Tregs are able to induce the senescent phenotype of T cells [12, 13]. For instance, Ye et al. [12] demonstrated that human Treg cells induced the senescent phenotype of both naïve and effector T cells. The senescent state was induced through the p38 and ERK1/2 signaling pathways which increased the expression of p16, p21, and p53, i.e., the cell-cycle inhibitors which are common markers of senescent cells. Senescent T cells displayed a strong downregulation in the expression of costimulatory CD27 and CD28 proteins, whereas the secretion of pro-inflammatory factors, such as IL-6 and TNF-α, was enhanced indicating the presence of the SASP state. Surprisingly, senescent T cells showed an upregulation in the secretion of anti-inflammatory IL-10 and TGF-β cytokines which implies that senescent T cells are able to expand immunosenescence within tissues. Subsequently, Liu et al. [13] reported that human senescent T cells did not display the phenotype of exhausted T cells, and moreover, senescent T cells were different from the anergic T cells. They also revealed that the STAT1 and STAT3 signaling pathways were involved in the senescence process of T cells. In addition to the CD4^+^CD25^+^ Tregs, also human γδ Tregs were able to suppress the functions of naïve and effector T cells [130]. It seems that Tregs enhance not only the immunosenescence of T cells but also that of NK cells [131, 132].

### Immunosuppression prevents the immune surveillance by NK and CD8^+^ T cells

NK and CD8^+^ T cells are the major immune cells which perform immune surveillance within tissues and eliminate dysfunctional and injured cells. It is known that cytotoxic NK and CD8^+^ T cells recognize senescent cells and induce their death and clearance from tissues [32, 133]. However, the immunosenescence of NK and CD8^+^ T cells impairs the surveillance process and thus allowing the accumulation of senescent cells, both immune and non-immune cells, within tissues during the aging process [33]. There is substantial evidence that immunosuppressive cells, especially M-MDSCs and Tregs, inhibit the immunosurveillance capacity and cytotoxic activity of NK and CD8^+^ T cells [126, 131, 132]. To suppress the cytotoxicity of NK and CD8^+^ T cells, MDSCs and Tregs release soluble factors, such as NO, adenosine, and IDO. They can also form the contact-dependent interaction with NK cells through the membrane-bound TGF-β and the activating NKp30 receptors [126, 132, 134]. For instance, Li et al. [134] demonstrated that mouse MDSCs inhibited the expression of NKG2D receptors and the cytotoxic activity of NK cells through their membrane-bound TGF-β1 proteins. It seems that MDSCs inhibit the function of NK cells through the blocking of NKp30 (NCR3) and NKG2D receptors, two killer receptors present in NK cells. Tregs also inhibit the function of NK cells via the TGF-β/NKG2D complex [132]. Thus, it seems that the MDSC/Treg-induced immunosenescence of NK cells impairs the clearance of senescent cells, tumor cells, and many pathogens with aging as well as in inflammatory conditions.

## Signaling mechanisms involved in immunosuppression-induced immunosenescence

When examining the characteristics of the senescent state of non-immune (called here cellular senescence) and immune (immunosenescence) cells (Table 1), it seems that similar mechanisms exist in the regulation of senescence, although there might be some immune-specific mechanisms. There is more research data from the mechanisms leading to cellular senescence than that available about immunosenescence. It is known that cellular senescence is commonly associated with extrinsic and intrinsic damages, whereas the plasticity of immune cells might affect the senescence of immune cells. The arrest of the cell-cycle is the major phenotype of both cellular senescence and immunosenescence which is attributed to the activation of cyclin-dependent kinase inhibitors (CDKI), e.g., p16, p21, and p53 (Table 1). There are multiple insults which can arrest the cell-cycle, such as DNA damage, amino acid deficiency, and many soluble factors. For instance, TGF-β is a potent inhibitor of cellular proliferation and an inducer of cellular senescence [30, 100]. Delisle et al. [135] demonstrated that TGF-β activated Smad3 signaling in mouse CD4^+^ T cells and inhibited the responses mediated through the CD28-dependent signaling, e.g., the growth and proliferation of T cells. Accordingly, Tiemessen et al. [136] revealed that TGF-β administration inhibited the cell cycle progression of human antigen-specific CD4^+^ T cells. Interestingly, TGF-β did not affect the production of cytokines indicating that the regulation of cell cycle arrest and the pro-inflammatory SASP are separated. Viel et al. [137] reported that TGF-β repressed the activity of mTOR in human NK cells, inhibiting their proliferation and reducing their cytotoxicity. It is known that Smad3 signaling stimulates the expression of CDKIs and subsequently prevents cell proliferation and induces cellular senescence [100].

There is convincing evidence that p38 mitogen-activated protein kinase (p38MAPK) has a crucial role in the generation of cellular senescence and immunosenescence [69, 138, 139]. A diverse set of insults associated with immunosuppression, e.g., TGF-β and IL-10 as well as oxidative (ROS/RNS) and endoplasmic reticulum (ER) stresses, can activate p38MAPK signaling which in downstream activates several protein kinases, transcription factors, and other regulatory proteins. For instance, p38MAPK is an important Smad-independent target of TGF-β signaling [140]. The activation of p38MAPK has been linked to cell-cycle arrest, DNA damage, telomere shortening, SASP induction, and autophagy inhibition in senescent immune cells [69, 139, 141, 142], all of which are hallmarks of immunosenescence (Table 1). Interestingly, Ding et al. [143] reported that the post-traumatic (trauma/hemorrhage) immunosuppression impaired mouse lung immunity against pneumococcal pneumonia. They revealed that an increase in the activity of p38MAPK reduced the phagocytosis of lung macrophages and impaired the killing of *Streptococcus pneumoniae* in post-traumatic mice. The exposure with p38MAPK inhibitor prevented these immune disturbances and improved mouse survival. Accordingly, Lanna et al. [142] demonstrated that sestrins, p53-inducible stress-sensing proteins, were able to bind to ERK, JNK, and p38MAPK enzymes and to establish the formation of a sestrin-MAPK activation complex (sMAC) in mouse and human T cells. The expression of sestrins displayed a tenfold increase in CD4^+^ T cells of elderly subjects as compared to younger individuals. The formation of the sMAC complexes was also significantly increased in human T cells with aging. They reported that the activation of sMAC kinases promoted T cell senescence, but each of the MAPKs controlled unique properties of senescence, e.g., p38MAPK inhibited telomerase activity and evoked telomere shortening. In addition to telomerase inhibition, Lanna et al. [139] reported that the activation of p38MAPK also inhibited human T cell proliferation, induced DNA damages, and disturbed the function of the TCR signalosome.

The TGF-β signaling pathways as well as the activation of p38MAPK are also important inducers of the SASP state which is associated with cellular senescence and immunosenescence [69, 72, 144]. Signaling through both TGF-β and p38MAPK can activate the nuclear factor-κB (NF-κB) system which is the major inducer of the genes involved in the SASP secretome [72]. Freund et al. [144] reported that p38MAPK regulated the pro-inflammatory SASP secretome via the activation of NF-κB signaling in human fibroblasts. Accordingly, Callender et al. [69] demonstrated that the activation of p38MAPK induced the SASP state in human senescent CD8^+^ T cells. Wang et al. [55] reported that LPS treatment for 24 h of mouse macrophages and human peripheral blood mononuclear cells (PBMC) evoked a senescent state through the NF-κB-induced activation of bromodomain-containing protein 4 (BRD4), an epigenetic regulator of gene transactivation. Senescent macrophages displayed a clear SASP state, and moreover, they showed an increased lipid uptake, a process associated with atherosclerosis. The inhibition of BRD4 prevented the senescence of mouse macrophages and lipid accumulation into senescent macrophages. It does seem that immunosenescence is under epigenetic regulation (see below).

Amino acid catabolism is a potent immunosuppressive mechanism since many effector immune cells are auxotropic and thus are unable to synthesize several amino acids, e.g., L-arginine and tryptophan [145]. Immunosuppressive cells express and secrete ARG1 and IDO enzymes which diminish the amounts of L-arginine and tryptophan, respectively, from the inflammatory microenvironment. The shortage of amino acids stimulates the expression of general control nonderepressible 2 (GCN2), a stress-kinase which activates eIF2α kinase and ATF4 transcription factor [146]. Subsequently, eIF2α and ATF4 inhibit protein synthesis and the proliferation of immune cells in inflammatory sites. It is known that a deficiency of amino acids not only inhibits the proliferation of T cells, but it also interferes with their TCR and co-stimulatory mechanisms and consequently can induce the anergy of T cells [147]. IDO catabolizes tryptophan into kynurenine and a number of other active metabolites. The kynurenine pathway is a powerful immunoregulation mechanism, i.e., it inhibits the functions of T and NK cells, whereas it activates Tregs and MDSCs [148, 149]. For instance, the kynurenine pathway induced the exhaustion of human CD4^+^ T cells [150] and provoked the selective apoptosis of mouse Th1 cells [151]. Della Chiesa et al. [152] demonstrated that kynurenine exposure impaired the expression of the activating NKp30 and NKG2D receptors of human NK cells. NKp30 and NKG2D receptors are important immune surveillance receptors, and thus kynurenine inhibited the immune recognition and clearance process performed by NK cells.

Interestingly, Mezrich et al. [153] demonstrated that kynurenine activated the aryl hydrocarbon receptor (AhR) which induced the differentiation of mouse naïve T cells into the FoxP3-positive Tregs. They also reported that TGF-β upregulated the expression of AhR in mouse T cells and thus enhanced their immunosuppressive activity. Subsequently, there has been intensive research activity to reveal the role of the IDO/kynurenine/AhR axis in pathological processes. For instance, it was claimed that kynurenine inhibited autophagy and promoted cellular senescence in mouse mesenchymal stem cells through the AhR pathway [154]. The aging process is associated with an increase in the expression of IDO and the enhanced production of kynurenine [155]. In particular, the kynurenine pathway is activated in chronic inflammatory conditions, such as atherosclerosis [156]. It is known that the kynurenine-mediated activation of AhR is a strong stimulating signal for the generation of Tregs in mice, whereas the blockade of the activity of AhR restricted the immunosuppression induced by kynurenine [157]. Moreover, Neamah et al. [158] demonstrated that the activation of AhR with the environmental toxin 2,3,7,8-tetrachlorodibenzo-p-dioxin (TCDD) in mice induced a massive chemokine induction which promoted the mobilization of both M-MDSCs and PMN-MDSCs from the bone marrow to the peritoneal cavity. The TCDD-induced MDSCs robustly suppressed the activation of T cells and also reduced the inflammation after Con A-induced hepatitis. Although the expression of AhR is low in many subsets of lymphocytes, the activation of AhR was able to suppress the differentiation of B cells and reduce their antibody production [159]. It seems that amino acid deprivation stimulates extensive immunosuppression through the activation of Tregs and MDSCs which subsequently suppress effector immune cells and provoke immunosenescence through the secretion of anti-inflammatory cytokines.

## Immunosuppressive factors are epigenetic masters of immunosenescent state

There is substantial evidence that epigenetic mechanisms control the cell lineage commitments and subsequently cell-type specific differentiation and maturation processes [160]. However, the chromatin landscape of mature cells can be reprogrammed into different cellular phenotypes; especially, immune cells display a significant plasticity which is dependent on epigenetic remodelling [161]. There are several studies indicating that aging processes as well as the cellular senescence state are associated with robust changes in the chromatin landscape and transcription signature [162, 163]. It is known that epigenetic mechanisms are involved in the generation of both chronic inflammatory and the SASP states [164, 165]. The senescence-associated epigenetic changes in the chromatin landscape include (i) increased formation of heterochromatin domains (SAHF), (ii) global DNA hypomethylation concurrently with focal hypermethylation, (iii) repressive histone modifications, (iv) altered nucleosomal compositions, and (v) perturbations of the contacts between nuclear lamina and chromatin structures [162]. For instance, Lyu et al. [166] reported that TGF-β signaling via Smad factors enhanced the accumulation of miR-29a/c which suppressed the expression of Suv4-20 h histone methyltransferase thus reducing the methylation of genomic H4K20 sites in human fibroblasts. This loss of H4K20 methylation disturbed the DNA repair processes and promoted cellular senescence and accelerated cardiac aging in mice.

The mechanisms of epigenetic regulation of immunosenescence are still not as broadly understood as those of cellular senescence. However, the major signaling pathways inducing immunosenescence involve the TGF-β/SMAD and the IL-10/STAT3 pathways which are known to control the activity of several important epigenetic regulators, e.g., DNA methyltransferases and histone demethylases [164, 167]. In addition, the expression of *TGF-β1*, *IL-10*, *AhR*, and *IDO1* genes are the subject of complex epigenetic regulation [168–171]. Epigenetic mechanisms also regulate the activity of the immunosuppressive network, e.g., by controlling the expression of FoxP3 gene, the master gene of Tregs [172]. In addition, TGF-β exposure was reported to reduce the immune surveillance activity of mouse and human NK cells via epigenetic regulation [98]. By exploiting omics techniques, Zhao et al. [173] demonstrated that there are significant changes in the transcriptomes, miRNAomes, and DNA methylomes of human CD4^+^ T cells during the aging process. Their results indicated that the expression levels of many age- and immune-related genes were under the regulation of DNA methylation. Moreover, Sidler et al. [174] reported that the aging process evoked significant changes in epigenetic regulation and gene expression in rat thymus and spleen which could induce thymic involution and peripheral immunosenescence. The epigenetic hallmarks of T cell aging include a global decrease in DNA methylation, whereas there is an increase in the number of heterogeneous loci of methylated CpG [175]. These scattered DNA methylation sites have been assessed as an epigenetic clock which can be used to evaluate the difference between the epigenetic age and the chronological age [176]. Many inflammatory diseases associated with increased immunosenescence, such as HIV infections, accelerate the epigenetic clock [177].

## Future perspectives: to rejuvenate or to prevent immunosuppression?

Currently, there is intense research activity attempting to understand whether it is possible to rejuvenate senescent cells or even cleanse them via senolysis from aged tissues. A plethora of mechanisms have been proposed as ways to inhibit age-related senescence and thus delay the aging process [163, 178, 179]. It seems that the prevention of those signaling pathways leading to the senescent state or the reprogramming of the epigenetic landscape might maintain the efficiency of the immune system with aging and pathological conditions. There are several experimental studies indicating that the blockage of the distinct signaling pathways associated with immunosenescence would be able to alleviate the immune deficiency, e.g., the inhibition of p38MAPK signaling [180], the inhibition of phosphoinositide 3-kinase activity [181], and the downregulation of inhibitory SHP-1 phosphatase [182]. Moreover, many experimental approaches have been exploited to attempt to rejuvenate immunosenescent T cells in elderly individuals, e.g., through the administration of certain interleukins, growth factors, checkpoint inhibitors, and diets as well as exposure to extracellular vesicles obtained from the serum of young donors [183, 184]. Moreover, Keren et al. [185] demonstrated that the depletion of B cells from old mice stimulated B cell lymphopoiesis in their bone marrow and rejuvenated the peripheral B cell lineage. More recently, some attempts have been made to turn epigenetic clocks backwards in immunosenescent cells [186]. For instance, the inhibition of the bromodomain protein BRD4 with JQ-1 and I-BET762 prevented the LPS-induced senescence of human THP-1 macrophages [55]. This treatment downregulated the expression of p16, p21, and p53, reduced the number of SA-β-gal-positive cells, and inhibited the secretion of SASP cytokines and chemokines. However, there are a number of challenges to be overcome, e.g., in the targeting and safety of these drugs before epidrugs can be used in epigenetic therapy.

Given that increased immunosuppression evokes immunosenescence, it seems pointless to rejuvenate immunosenescent cells with aging and in many inflammatory pathological conditions, since the enhanced immunosuppressive armament will prevent a permanent rejuvenation. The most reasonable therapeutic target would seem to be immunosuppressive cells, especially MDSCs and Tregs, which are able to support immunosuppression. It is known that immunosuppression is a major problem in cancer biology, thus different strategies have been exploited in attempts to inhibit the activities of MDSCs and Tregs as ways to improve the efficacy of immunotherapy [187, 188]. For instance, the immunosuppressive functions of MDSCs have been inhibited by (i) normalizing their production in myelopoiesis, (ii) promoting their maturation to effector immune cells, (iii) reducing their trafficking and expansion, (iv) inhibiting their immunosuppressive activities, and (v) depleting their presence in tumors. There are several pharmacological compounds which have been demonstrated to inhibit the immunosuppressive activity of MDSCs, e.g., many phytochemicals suppress the activity of MDSCs [189]. Interestingly, the agonists binding to TLR7 and TLR8, e.g., resiquimod, promote the differentiation of MDSCs into macrophages and dendritic cells [190]. Considering that Tregs maintain the immune tolerance of the body, their inhibition will demand more specific mechanisms in order to avoid the generation of autoimmune diseases. There are many approaches which could be employed to inhibit the functions of Tregs, e.g., (i) targeting their immune checkpoint receptors, (ii) skewing them towards anti-tumor T cell phenotypes, (iii) targeting specific Treg proteins, such as FoxP3, and (iv) inhibiting their metabolism [188]. Moreover, given that immunosuppressive cells generate adenosine, there are studies indicating that the inhibition of the adenosinergic pathway was able to rejuvenate innate and adaptive immunity in tumors [191]. Many anti-aging compounds, e.g., metformin, have been shown to inhibit the functions of MDSCs and Tregs [192, 193]. Currently, it is not known whether these compounds will be able to prevent immunosenescence by inhibiting the immunosuppressive network.

## References

[1] Franceschi C, Valensin S, Bonafe M, Paolisso G, Yashin AI, Monti D, De Benedictis G (2000). The network and the remodeling theories of aging: historical background and new perspectives. Exp Gerontol.

[2] Xu W, Wong G, Hwang YY, Larbi A (2020). The untwining of immunosenescence and aging. Semin Immunopathol.

[3] Krenzien F, ElKhal A, Quante M, Rodriguez Cetina Biefer H, Hirofumi U, Gabardi S, Tullius SG (2015). A rationale for age-adapted immunosuppression in organ transplantation. Transplantation.

[4] Monneret G, Gossez M, Venet F (2019). Sepsis and immunosenescence: closely associated in a vicious circle. Aging Clin Exp Res.

[5] Lian J, Yue Y, Yu W, Zhang Y (2020). Immunosenescence: a key player in cancer development. J Hematol Oncol.

[6] Hernandez-Segura A, Nehme J, Demaria M (2018). Hallmarks of cellular senescence. Trends Cell Biol.

[7] Gentile LF, Cuenca AG, Efron PA, Ang D, Bihorac A, McKinley BA, Moldawer LL, Moore FA (2012). Persistent inflammation and immunosuppression: a common syndrome and new horizon for surgical intensive care. J Trauma Acute Care Surg.

[8] Kanterman J, Sade-Feldman M, Baniyash M (2012). New insights into chronic inflammation-induced immunosuppression. Semin Cancer Biol.

[9] Amodio G, Cichy J, Conde P, Matteoli G, Moreau A, Ochando J, Oral BH, Pekarova M, Ryan EJ, Roth J (2019). Role of myeloid regulatory cells (MRCs) in maintaining tissue homeostasis and promoting tolerance in autoimmunity, inflammatory disease and transplantation. Cancer Immunol Immunother.

[10] Gabrilovich DI, Nagaraj S (2009). Myeloid-derived suppressor cells as regulators of the immune system. Nat Rev Immunol.

[11] Pawelec G (2020). The human immunosenescence phenotype: does it exist?. Semin Immunopathol.

[12] Ye J, Huang X, Hsueh EC, Zhang Q, Ma C, Zhang Y, Varvares MA, Hoft DF, Peng G (2012). Human regulatory T cells induce T-lymphocyte senescence. Blood.

[13] Liu X, Mo W, Ye J, Li L, Zhang Y, Hsueh EC, Hoft DF, Peng G (2018). Regulatory T cells trigger effector T cell DNA damage and senescence caused by metabolic competition. Nat Commun.

[14] Gayoso I, Sanchez-Correa B, Campos C, Alonso C, Pera A, Casado JG, Morgado S, Tarazona R, Solana R (2011). Immunosenescence of human natural killer cells. J Innate Immun.

[15] Fulop T, Dupuis G, Witkowski JM, Larbi A (2016). The role of immunosenescence in the development of age-related diseases. Rev Invest Clin.

[16] Barbe-Tuana F, Funchal G, Schmitz CRR, Maurmann RM, Bauer ME (2020). The interplay between immunosenescence and age-related diseases. Semin Immunopathol.

[17] Fulop T, Larbi A, Dupuis G, Le Page A, Frost EH, Cohen AA, Witkowski JM, Franceschi C (2018). Immunosenescence and inflamm-aging as two sides of the same coin: friends or foes?. Front Immunol.

[18] Akbar AN, Henson SM (2011). Are senescence and exhaustion intertwined or unrelated processes that compromise immunity?. Nat Rev Immunol.

[19] Zhao Y, Shao Q, Peng G (2020). Exhaustion and senescence: two crucial dysfunctional states of T cells in the tumor microenvironment. Cell Mol Immunol.

[20] Wang S, Zhang Q, Hui H, Agrawal K, Karris MAY, Rana TM (2020). An atlas of immune cell exhaustion in HIV-infected individuals revealed by single-cell transcriptomics. Emerg Microbes Infect.

[21] Lee J, Ahn E, Kissick HT, Ahmed R (2015). Reinvigorating exhausted T cells by blockade of the PD-1 pathway. For Immunopathol Dis Therap.

[22] Judge SJ, Murphy WJ, Canter RJ (2020). Characterizing the dysfunctional NK cell: assessing the clinical relevance of exhaustion, anergy, and senescence. Front Cell Infect Microbiol.

[23] Pawelec G, Remarque E, Barnett Y, Solana R (1998). T cells and aging. Front Biosci.

[24] Salminen A, Kaarniranta K, Kauppinen A (2019). Immunosenescence: the potential role of myeloid-derived suppressor cells (MDSC) in age-related immune deficiency. Cell Mol Life Sci.

[25] Rodriguez IJ, Lalinde Ruiz N, Llano Leon M, Martínez Enriquez L, Montilla Velasquez MDP, Ortiz Aguirre JP, Rodriguez Bohorquez OM, Velandia Vargas EA, Hernandez ED, Parra Lopez CA (2021) Immunosenescence study of T cells: a systematic review. Front Immunol 11:604591 10.3389/fimmu.2020.604591 PMC7843425 33519813

[26] Vallejo AN (2007). Immune remodeling: lessons from repertoire alterations during chronological aging and in immune-mediated disease. Trends Mol Med.

[27] Pang WW, Price EA, Sahoo D, Beerman I, Maloney WJ, Rossi DJ, Schrier SL, Weissman IL (2011). Human bone marrow hematopoietic stem cells are increased in frequency and myeloid-biased with age. Proc Natl Acad Sci U S A.

[28] Egorov ES, Kasatskaya SA, Zubov VN, Izraelson M, Nakonechnaya TO, Staroverov DB, Angius A, Cucca F, Mamedov IZ, Rosati E (2018). The changing landscape of naive T cell receptor repertoire with human aging. Front Immunol.

[29] Kennedy DE, Knight KL (2015). Inhibition of B lymphopoiesis by adipocytes and IL-1-producing myeloid-derived suppressor cells. J Immunol.

[30] Li MO, Wan YY, Sanjabi S, Robertson AK, Flavell RA (2006). Transforming growth factor-β regulation of immune responses. Annu Rev Immunol.

[31] Frasca D, Diaz A, Romero M, Garcia D, Blomberg BB (2020). B cell immunosenescence. Annu Rev Cell Dev Biol.

[32] Sagiv A, Burton DG, Moshayev Z, Vadai E, Wensveen F, Ben-Dor S, Golani O, Polic B, Krizhanovsky V (2016). NKG2D ligands mediate immunosurveillance of senescent cells. Aging (Albany NY).

[33] Salminen A (2021) Feed-forward regulation between cellular senescence and immunosuppression promotes the aging process and age-related diseases. Ageing Res Rev 67:101280 10.1016/j.arr.2021.101280 33581314

[34] Hazeldine J, Lord JM (2013). The impact of ageing on natural killer cell function and potential consequences for health in older adults. Ageing Res Rev.

[35] Almeida-Oliveira A, Smith-Carvalho M, Porto LC, Cardoso-Oliveira J, Ribeiro Ados S, Falcao RR, Abdelhay E, Bouzas LF, Thuler LC, Ornellas MH (2011). Age-related changes in natural killer cell receptors from childhood through old age. Hum Immunol.

[36] Kared H, Martelli S, Ng TP, Pender SL, Larbi A (2016). CD57 in human natural killer cells and T-lymphocytes. Cancer Immunol Immunother.

[37] Hazeldine J, Hampson P, Lord JM (2012). Reduced release and binding of perforin at the immunological synapse underlies the age-related decline in natural killer cell cytotoxicity. Aging Cell.

[38] Rajagopalan S, Long EO (2012). Cellular senescence induced by CD158d reprograms natural killer cells to promote vascular remodeling. Proc Natl Acad Sci U S A.

[39] Stout RD, Suttles J (2005). Immunosenescence and macrophage functional plasticity: dysregulation of macrophage function by age-associated microenvironmental changes. Immunol Rev.

[40] Bhushan M, Cumberbatch M, Dearman RJ, Andrew SM, Kimber I, Griffiths CE (2002). Tumour necrosis factor-α-induced migration of human Langerhans cells: the influence of ageing. Br J Dermatol.

[41] Agrawal A, Gupta S (2011). Impact of aging on dendritic cell functions in humans. Ageing Res Rev.

[42] Chougnet CA, Thacker RI, Shehata HM, Hennies CM, Lehn MA, Lages CS, Janssen EM (2015). Loss of phagocytic and antigen cross-presenting capacity in aging dendritic cells is associated with mitochondrial dysfunction. J Immunol.

[43] Panda A, Qian F, Mohanty S, van Duin D, Newman FK, Zhang L, Chen S, Towle V, Belshe RB, Fikrig E (2010). Age-associated decrease in TLR function in primary human dendritic cells predicts influenza vaccine response. J Immunol.

[44] Linehan E, Fitzgerald DC (2015). Ageing and the immune system: focus on macrophages. Eur J Microbiol Immunol (Bp).

[45] De Maeyer RPH, Chambers ES (2021). The impact of ageing on monocytes and macrophages. Immunol Lett.

[46] Kohut ML, Senchina DS, Madden KS, Martin AE, Felten DL, Moynihan JA (2004). Age effects on macrophage function vary by tissue site, nature of stimulant, and exercise behavior. Exp Gerontol.

[47] Akbar AN, Henson SM, Lanna A (2016). Senescence of T lymphocytes: implications for enhancing human immunity. Trends Immunol.

[48] Behmoaras J, Gil J (2021) Similarities and interplay between senescent cells and macrophages. J Cell Biol 220:e202010162 10.1083/jcb.202010162 PMC7769159 33355620

[49] Liu Y, Sanoff HK, Cho H, Burd CE, Torrice C, Ibrahim JG, Thomas NE, Sharpless NE (2009). Expression of p16(INK4a) in peripheral blood T-cells is a biomarker of human aging. Aging Cell.

[50] Onyema OO, Njemini R, Bautmans I, Renmans W, De Waele M, Mets T (2012). Cellular aging and senescence characteristics of human T-lymphocytes. Biogerontology.

[51] Mondal AM, Horikawa I, Pine SR, Fujita K, Morgan KM, Vera E, Mazur SJ, Appella E, Vojtesek B, Blasco MA (2013). p53 isoforms regulate aging- and tumor-associated replicative senescence in T lymphocytes. J Clin Invest.

[52] Rajagopalan S, Lee EC, DuPrie ML, Long EO (2014). TNFR-associated factor 6 and TGF-β-activated kinase 1 control signals for a senescence response by an endosomal NK cell receptor. J Immunol.

[53] Hall BM, Balan V, Gleiberman AS, Strom E, Krasnov P, Virtuoso LP, Rydkina E, Vujcic S, Balan K, Gitlin I (2016). Aging of mice is associated with p16(Ink4a)- and β-galactosidase-positive macrophage accumulation that can be induced in young mice by senescent cells. Aging (Albany NY).

[54] Liu JY, Souroullas GP, Diekman BO, Krishnamurthy J, Hall BM, Sorrentino JA, Parker JS, Sessions GA, Gudkov AV, Sharpless NE (2019). Cells exhibiting strong p16INK4a promoter activation *in vivo* display features of senescence. Proc Natl Acad Sci U S A.

[55] Wang H, Fu H, Zhu R, Wu X, Ji X, Li X, Jiang H, Lin Z, Tang X, Sun S (2020). BRD4 contributes to LPS-induced macrophage senescence and promotes progression of atherosclerosis-associated lipid uptake. Aging (Albany NY).

[56] Gerland LM, Genestier L, Peyrol S, Michallet MC, Hayette S, Urbanowicz I, Ffrench P, Magaud JP, Ffrench M (2004). Autolysosomes accumulate during in vitro CD8^+^ T-lymphocyte aging and may participate in induced death sensitization of senescent cells. Exp Gerontol.

[57] Covre LP, Martins RF, Devine OP, Chambers ES, Vukmanovic-Stejic M, Silva JA, Dietze R, Rodrigues RR, de Matos Guedes HL, Falqueto A (2019). Circulating senescent T cells are linked to systemic inflammation and lesion size during human cutaneous Leishmaniasis. Front Immunol.

[58] Kaszubowska L (2008). Telomere shortening and ageing of the immune system. J Physiol Pharmacol.

[59] Sebastian C, Herrero C, Serra M, Lloberas J, Blasco MA, Celada A (2009). Telomere shortening and oxidative stress in aged macrophages results in impaired STAT5a phosphorylation. J Immunol.

[60] Sanderson SL, Simon AK (2017). In aged primary T cells, mitochondrial stress contributes to telomere attrition measured by a novel imaging flow cytometry assay. Aging Cell.

[61] Stranks AJ, Hansen AL, Panse I, Mortensen M, Ferguson DJ, Puleston DJ, Shenderov K, Watson AS, Veldhoen M, Phadwal K (2015). Autophagy controls acquisition of aging features in macrophages. J Innate Immun.

[62] Zhang H, Puleston DJ, Simon AK (2016). Autophagy and immune senescence. Trends Mol Med.

[63] Bektas A, Schurman SH, Gonzalez-Freire M, Dunn CA, Singh AK, Macian F, Cuervo AM, Sen R, Ferrucci L (2019). Age-associated changes in human CD4^+^ T cells point to mitochondrial dysfunction consequent to impaired autophagy. Aging (Albany NY).

[64] Hurst KE, Lawrence KA, Essman MT, Walton ZJ, Leddy LR, Thaxton JE (2019). Endoplasmic reticulum stress contributes to mitochondrial exhaustion of CD8^+^ T cells. Cancer Immunol Res.

[65] Sukhorukov VN, Khotina VA, Bagheri Ekta M, Ivanova EA, Sobenin IA, Orekhov AN (2020). Endoplasmic reticulum stress in macrophages: the vicious circle of lipid accumulation and pro-inflammatory response. Biomedicines.

[66] Kannan S, Dawany N, Kurupati R, Showe LC, Ertl HC (2016). Age-related changes in the transcriptome of antibody-secreting cells. Oncotarget.

[67] Vida C, de Toda IM, Cruces J, Garrido A, Gonzalez-Sanchez M, De la Fuente M (2017). Role of macrophages in age-related oxidative stress and lipofuscin accumulation in mice. Redox Biol.

[68] Garrido A, Cruces J, Ceprian N, Vara E, de la Fuente M (2019). Oxidative-inflammatory stress in immune cells from adult mice with premature aging. Int J Mol Sci.

[69] Martinez de Toda I, Vida C, Sanz San Miguel L, De la Fuente M (2019). Function, oxidative, and inflammatory stress parameters in immune cells as predictive markers of lifespan throughout aging. Oxid Med Cell Longev.

[70] Callender LA, Carroll EC, Beal RWJ, Chambers ES, Nourshargh S, Akbar AN, Henson SM (2018) Human CD8^+^ EMRA T cells display a senescence-associated secretory phenotype regulated by p38 MAPK. Aging Cell 17:e12675 10.1111/acel.12675 PMC5770853 29024417

[71] Frasca D, Diaz A, Romero M, Blomberg BB (2017). Human peripheral late/exhausted memory B cells express a senescent-associated secretory phenotype and preferentially utilize metabolic signaling pathways. Exp Gerontol.

[72] Salminen A, Kauppinen A, Kaarniranta K (2012). Emerging role of NF-κB signaling in the induction of senescence-associated secretory phenotype (SASP). Cell Signal.

[73] Kaltschmidt B, Kaltschmidt C, Hofmann TG, Hehner SP, Dröge W, Schmitz ML (2000). The pro- or anti-apoptotic function of NF-κB is determined by the nature of the apoptotic stimulus. Eur J Biochem.

[74] Salminen A, Ojala J, Kaarniranta K (2011). Apoptosis and aging: increased resistance to apoptosis enhances the aging process. Cell Mol Life Sci.

[75] Spaulding C, Guo W, Effros RB (1999). Resistance to apoptosis in human CD8^+^ T cells that reach replicative senescence after multiple rounds of antigen-specific proliferation. Exp Gerontol.

[76] Dennett NS, Barcia RN, McLeod JD (2002). Age associated decline in CD25 and CD28 expression correlate with an increased susceptibility to CD95 mediated apoptosis in T cells. Exp Gerontol.

[77] Chong Y, Ikematsu H, Yamaji K, Nishimura M, Nabeshima S, Kashiwagi S, Hayashi J (2005). CD27^+^ (memory) B cell decrease and apoptosis-resistant CD27^-^ (naive) B cell increase in aged humans: implications for age-related peripheral B cell developmental disturbances. Int Immunol.

[78] Bauer ME (2020). Accelerated immunosenescence in rheumatoid arthritis: impact on clinical progression. Immun Ageing.

[79] Weyand CM, Yang Z, Goronzy JJ (2014). T-cell aging in rheumatoid arthritis. Curr Opin Rheumatol.

[80] Cunha LL, Perazzio SF, Azzi J, Cravedi P, Riella LV (2020). Remodeling of the immune response with aging: immunosenescence and its potential impact on COVID-19 immune response. Front Immunol.

[81] Shirakawa K, Yan X, Shinmura K, Endo J, Kataoka M, Katsumata Y, Yamamoto T, Anzai A, Isobe S, Yoshida N (2016). Obesity accelerates T cell senescence in murine visceral adipose tissue. J Clin Invest.

[82] Bauer ME, Teixeira AL (2019). Inflammation in psychiatric disorders: what comes first?. Ann NY Acad Sci.

[83] Bauer ME, Teixeira AL (2021). Neuroinflammation in mood disorders: role of regulatory immune cells. NeuroImmunoModulation.

[84] Gelson W, Hoare M, Vowler S, Shankar A, Gibbs P, Akbar AN, Alexander GJ (2010). Features of immune senescence in liver transplant recipients with established grafts. Liver Transpl.

[85] Lecot P, Alimirah F, Desprez PY, Campisi J, Wiley C (2016). Context-dependent effects of cellular senescence in cancer development. Br J Cancer.

[86] Sanchez-Correa B, Campos C, Pera A, Bergua JM, Arcos MJ, Banas H, Casado JG, Morgado S, Duran E, Solana R (2016). Natural killer cell immunosenescence in acute myeloid leukaemia patients: new targets for immunotherapeutic strategies?. Cancer Immunol Immunother.

[87] Wang D, DuBois RN (2015). Immunosuppression associated with chronic inflammation in the tumor microenvironment. Carcinogenesis.

[88] Ferrer IR, Hester J, Bushell A, Wood KJ (2014). Induction of transplantation tolerance through regulatory cells: from mice to men. Immunol Rev.

[89] Sacchi A, Grassi G, Bordoni V, Lorenzini P, Cimini E, Casetti R, Tartaglia E, Marchioni L, Petrosillo N, Palmieri F (2020). Early expansion of myeloid-derived suppressor cells inhibits SARS-CoV-2 specific T-cell response and may predict fatal COVID-19 outcome. Cell Death Dis.

[90] Mira JC, Brakenridge SC, Moldawer LL, Moore FA (2017). Persistent inflammation, immunosuppression and catabolism syndrome. Crit Care Clin.

[91] Lindau D, Gielen P, Kroesen M, Wesseling P, Adema GJ (2013). The immunosuppressive tumour network: myeloid-derived suppressor cells, regulatory T cells and natural killer T cells. Immunology.

[92] Salminen A (2020) Activation of immunosuppressive network in the aging process. Ageing Res Rev 57:100998 10.1016/j.arr.2019.100998 31838128

[93] Nagaraj S, Youn JI, Gabrilovich DI (2013). Reciprocal relationship between myeloid-derived suppressor cells and T cells. J Immunol.

[94] Park YJ, Song B, Kim YS, Kim EK, Lee JM, Lee GE, Kim JO, Kim YJ, Chang WS, Kang CY (2013). Tumor microenvironmental conversion of natural killer cells into myeloid-derived suppressor cells. Cancer Res.

[95] Sorokin L (2010). The impact of the extracellular matrix on inflammation. Nat Rev Immunol.

[96] Ouyang W, Rutz S, Crellin NK, Valdez PA, Hymowitz SG (2011). Regulation and functions of the IL-10 family of cytokines in inflammation and disease. Annu Rev Immunol.

[97] Whiteside TL, Jackson EK (2013). Adenosine and prostaglandin e2 production by human inducible regulatory T cells in health and disease. Front Immunol.

[98] Salminen A (2021). Increased immunosuppression impairs tissue homeostasis with aging and age-related diseases. J Mol Med (Berl).

[99] Regis S, Dondero A, Caliendo F, Bottino C, Castriconi R (2020). NK cell function regulation by TGF-β-induced epigenetic mechanisms. Front Immunol.

[100] Tominaga K, Suzuki HI (2019). TGF-β signaling in cellular senescence and aging-related pathology. Int J Mol Sci.

[101] Nakamura K, Matsunaga K (1998). Susceptibility of natural killer (NK) cells to reactive oxygen species (ROS) and their restoration by the mimics of superoxide dismutase (SOD). Cancer Biother Radiopharm.

[102] Nagaraj S, Gupta K, Pisarev V, Kinarsky L, Sherman S, Kang L, Herber DL, Schneck J, Gabrilovich DI (2007). Altered recognition of antigen is a mechanism of CD8^+^ T cell tolerance in cancer. Nat Med.

[103] Davalli P, Mitic T, Caporali A, Lauriola A, D'Arca D (2016). ROS, cell senescence, and novel molecular mechanisms in aging and age-related diseases. Oxid Med Cell Longev.

[104] Holt D, Ma X, Kundu N, Fulton A (2011). Prostaglandin E2 (PGE2) suppresses natural killer cell function primarily through the PGE2 receptor EP4. Cancer Immunol Immunother.

[105] Gomez I, Foudi N, Longrois D, Norel X (2013). The role of prostaglandin E2 in human vascular inflammation. Prostaglandins Leukot Essent Fatty Acids.

[106] Mackay IR (1972). Ageing and immunological function in man. Gerontologia.

[107] Roder JC, Duwe AK, Bell DA, Singhal SK (1978). Immunological senescence. I. The role of suppressor cells Immunology.

[108] Singhal SK, Roder JC, Duwe AK (1978). Suppressor cells in immunosenescence. Fed Proc.

[109] Gregg R, Smith CM, Clark FJ, Dunnion D, Khan N, Chakraverty R, Nayak L, Moss PA (2005). The number of human peripheral blood CD4^+^ CD25^high^ regulatory T cells increases with age. Clin Exp Immunol.

[110] Lages CS, Suffia I, Velilla PA, Huang B, Warshaw G, Hildeman DA, Belkaid Y, Chougnet C (2008). Functional regulatory T cells accumulate in aged hosts and promote chronic infectious disease reactivation. J Immunol.

[111] Sharma S, Dominguez AL, Lustgarten J (2006). High accumulation of T regulatory cells prevents the activation of immune responses in aged animals. J Immunol.

[112] Verschoor CP, Johnstone J, Millar J, Dorrington MG, Habibagahi M, Lelic A, Loeb M, Bramson JL, Bowdish DM (2013). Blood CD33^+^HLA^−^DR^−^ myeloid-derived suppressor cells are increased with age and a history of cancer. J Leukoc Biol.

[113] Enioutina EY, Bareyan D, Daynes RA (2011). A role for immature myeloid cells in immune senescence. J Immunol.

[114] Jackaman C, Radley-Crabb HG, Soffe Z, Shavlakadze T, Grounds MD, Nelson DJ (2013). Targeting macrophages rescues age-related immune deficiencies in C57BL/6J geriatric mice. Aging Cell.

[115] Wang Y, Wehling-Henricks M, Samengo G, Tidball JG (2015). Increases of M2a macrophages and fibrosis in aging muscle are influenced by bone marrow aging and negatively regulated by muscle-derived nitric oxide. Aging Cell.

[116] Agius E, Lacy KE, Vukmanovic-Stejic M, Jagger AL, Papageorgiou AP, Hall S, Reed JR, Curnow SJ, Fuentes-Duculan J, Buckley CD (2009). Decreased TNF-α synthesis by macrophages restricts cutaneous immunosurveillance by memory CD4^+^ T cells during aging. J Exp Med.

[117] Kalathookunnel Antony A, Lian Z, Wu H (2018). T cells in adipose tissue in aging. Front Immunol.

[118] Ruhland MK, Loza AJ, Capietto AH, Luo X, Knolhoff BL, Flanagan KC, Belt BA, Alspach E, Leahy K, Luo J (2016). Stromal senescence establishes an immunosuppressive microenvironment that drives tumorigenesis. Nat Commun.

[119] Tcyganov E, Mastio J, Chen E, Gabrilovich DI (2018). Plasticity of myeloid-derived suppressor cells in cancer. Curr Opin Immunol.

[120] Chen J, Ye Y, Liu P, Yu W, Wei F, Li H, Yu J (2017). Suppression of T cells by myeloid-derived suppressor cells in cancer. Hum Immunol.

[121] Özkan B, Lim H, Park SG (2018). Immunomodulatory function of myeloid-derived suppressor cells during B cell-mediated immune responses. Int J Mol Sci.

[122] Park MJ, Lee SH, Kim EK, Lee EJ, Baek JA, Park SH, Kwok SK, Cho ML (2018). Interleukin-10 produced by myeloid-derived suppressor cells is critical for the induction of Tregs and attenuation of rheumatoid inflammation in mice. Sci Rep.

[123] Park MJ, Lee SH, Kim EK, Lee EJ, Park SH, Kwok SK, Cho ML (2016). Myeloid-derived suppressor cells induce the expansion of regulatory B cells and ameliorate autoimmunity in the Sanroque mouse model of systemic lupus erythematosus. Arthritis Rheumatol.

[124] Poschke I, Mao Y, Adamson L, Salazar-Onfray F, Masucci G, Kiessling R (2012). Myeloid-derived suppressor cells impair the quality of dendritic cell vaccines. Cancer Immunol Immunother.

[125] Hu CE, Gan J, Zhang RD, Cheng YR, Huang GJ (2011). Up-regulated myeloid-derived suppressor cell contributes to hepatocellular carcinoma development by impairing dendritic cell function. Scand J Gastroenterol.

[126] Hoechst B, Voigtlaender T, Ormandy L, Gamrekelashvili J, Zhao F, Wedemeyer H, Lehner F, Manns MP, Greten TF, Korangy F (2009). Myeloid derived suppressor cells inhibit natural killer cells in patients with hepatocellular carcinoma via the NKp30 receptor. Hepatology.

[127] Fortin C, Huang X, Yang Y (2012). NK cell response to vaccinia virus is regulated by myeloid-derived suppressor cells. J Immunol.

[128] Ostrand-Rosenberg S, Sinha P, Beury DW, Clements VK (2012). Cross-talk between myeloid-derived suppressor cells (MDSC), macrophages, and dendritic cells enhances tumor-induced immune suppression. Semin Cancer Biol.

[129] Nguyen HH, Tran BT, Muller W, Jack RS (2012). IL-10 acts as a developmental switch guiding monocyte differentiation to macrophages during a murine peritoneal infection. J Immunol.

[130] Ye J, Ma C, Hsueh EC, Eickhoff CS, Zhang Y, Varvares MA, Hoft DF, Peng G (2013). Tumor-derived γδ regulatory T cells suppress innate and adaptive immunity through the induction of immunosenescence. J Immunol.

[131] Trzonkowski P, Szmit E, Mysliwska J, Mysliwski A (2006). CD4+CD25+ T regulatory cells inhibit cytotoxic activity of CTL and NK cells in humans-impact of immunosenescence. Clin Immunol.

[132] Ralainirina N, Poli A, Michel T, Poos L, Andres E, Hentges F, Zimmer J (2007). Control of NK cell functions by CD4^+^CD25^+^ regulatory T cells. J Leukoc Biol.

[133] Pereira BI, Devine OP, Vukmanovic-Stejic M, Chambers ES, Subramanian P, Patel N, Virasami A, Sebire NJ, Kinsler V, Valdovinos A (2019). Senescent cells evade immune clearance via HLA-E-mediated NK and CD8^+^ T cell inhibition. Nat Commun.

[134] Li H, Han Y, Guo Q, Zhang M, Cao X (2009). Cancer-expanded myeloid-derived suppressor cells induce anergy of NK cells through membrane-bound TGF-β1. J Immunol.

[135] Delisle JS, Giroux M, Boucher G, Landry JR, Hardy MP, Lemieux S, Jones RG, Wilhelm BT, Perreault C (2013). The TGF-β-Smad3 pathway inhibits CD28-dependent cell growth and proliferation of CD4 T cells. Genes Immun.

[136] Tiemessen MM, Kunzmann S, Schmidt-Weber CB, Garssen J, Bruijnzeel-Koomen CA, Knol EF, van Hoffen E (2003). Transforming growth factor-β inhibits human antigen-specific CD4^+^ T cell proliferation without modulating the cytokine response. Int Immunol.

[137] Viel S, Marcais A, Guimaraes FS, Loftus R, Rabilloud J, Grau M, Degouve S, Djebali S, Sanlaville A, Charrier E et al (2016) TGF-β inhibits the activation and functions of NK cells by repressing the mTOR pathway. Sci Signal 9:ra19 10.1126/scisignal.aad1884 26884601

[138] Iwasa H, Han J, Ishikawa F (2003). Mitogen-activated protein kinase p38 defines the common senescence-signalling pathway. Genes Cells.

[139] Lanna A, Henson SM, Escors D, Akbar AN (2014). The kinase p38 activated by the metabolic regulator AMPK and scaffold TAB1 drives the senescence of human T cells. Nat Immunol.

[140] Yu L, Hebert MC, Zhang YE (2002). TGF-β receptor-activated p38 MAP kinase mediates Smad-independent TGF-β responses. EMBO J.

[141] Henson SM, Lanna A, Riddell NE, Franzese O, Macaulay R, Griffiths SJ, Puleston DJ, Watson AS, Simon AK, Tooze SA (2014). p38 signaling inhibits mTORC1-independent autophagy in senescent human CD8 T cells. J Clin Invest.

[142] Lanna A, Gomes DC, Muller-Durovic B, McDonnell T, Escors D, Gilroy DW, Lee JH, Karin M, Akbar AN (2017). A sestrin-dependent Erk-Jnk-p38 MAPK activation complex inhibits immunity during aging. Nat Immunol.

[143] Ding N, Dahlke K, Janze AK, Mailer PC, Maus R, Bohling J, Welte T, Bauer M, Riedemann NC, Maus UA (2012). Role of p38 mitogen-activated protein kinase in posttraumatic immunosuppression in mice. J Trauma Acute Care Surg.

[144] Freund A, Patil CK, Campisi J (2011). p38MAPK is a novel DNA damage response-independent regulator of the senescence-associated secretory phenotype. EMBO J.

[145] Murray PJ (2016). Amino acid auxotrophy as a system of immunological control nodes. Nat Immunol.

[146] Castilho BA, Shanmugam R, Silva RC, Ramesh R, Himme BM, Sattlegger E (2014). Keeping the eIF2α kinase Gcn2 in check. Biochim Biophys Acta.

[147] Munn DH, Sharma MD, Baban B, Harding HP, Zhang Y, Ron D, Mellor AL (2005). GCN2 kinase in T cells mediates proliferative arrest and anergy induction in response to indoleamine 2,3-dioxygenase. Immunity.

[148] Mandi Y, Vecsei L (2012). The kynurenine system and immunoregulation. J Neural Transm (Vienna).

[149] Holmgaard RB, Zamarin D, Li Y, Gasmi B, Munn DH, Allison JP, Merghoub T, Wolchok JD (2015). Tumor-expressed IDO recruits and activates MDSCs in a Treg-dependent manner. Cell Rep.

[150] Rad Pour S, Morikawa H, Kiani NA, Yang M, Azimi A, Shafi G, Shang M, Baumgartner R, Ketelhuth DFJ, Kamleh MA (2019). Exhaustion of CD4^+^ T-cells mediated by the kynurenine pathway in melanoma. Sci Rep.

[151] Fallarino F, Grohmann U, Vacca C, Bianchi R, Orabona C, Spreca A, Fioretti MC, Puccetti P (2002). T cell apoptosis by tryptophan catabolism. Cell Death Differ.

[152] Della Chiesa M, Carlomagno S, Frumento G, Balsamo M, Cantoni C, Conte R, Moretta L, Moretta A, Vitale M (2006). The tryptophan catabolite L-kynurenine inhibits the surface expression of NKp46- and NKG2D-activating receptors and regulates NK-cell function. Blood.

[153] Mezrich JD, Fechner JH, Zhang X, Johnson BP, Burlingham WJ, Bradfield CA (2010). An interaction between kynurenine and the aryl hydrocarbon receptor can generate regulatory T cells. J Immunol.

[154] Kondrikov D, Elmansi A, Bragg RT, Mobley T, Barrett T, Eisa N, Kondrikova G, Schoeinlein P, Aguilar-Perez A, Shi XM et al (2020) Kynurenine inhibits autophagy and promotes senescence in aged bone marrow mesenchymal stem cells through the aryl hydrocarbon receptor pathway. Exp Gerontol 130:110805 10.1016/j.exger.2019.110805 PMC7861134 31812582

[155] Sorgdrager FJH, Naude PJW, Kema IP, Nollen EA, Deyn PP (2019). Tryptophan metabolism in inflammaging: from biomarker to therapeutic target. Front Immunol.

[156] Baumgartner R, Forteza MJ, Ketelhuth DFJ (2019) The interplay between cytokines and the kynurenine pathway in inflammation and atherosclerosis. Cytokine 122:154148 10.1016/j.cyto.2017.09.004 28899580

[157] Campesato LF, Budhu S, Tchaicha J, Weng CH, Gigoux M, Cohen IJ, Redmond D, Mangarin L, Pourpe S, Liu C (2020). Blockade of the AHR restricts a Treg-macrophage suppressive axis induced by L-kynurenine. Nat Commun.

[158] Neamah WH, Singh NP, Alghetaa H, Abdulla OA, Chatterjee S, Busbee PB, Nagarkatti M, Nagarkatti P (2019). AhR activation leads to massive mobilization of myeloid-derived suppressor cells with immunosuppressive activity through regulation of CXCR2 and microRNA miR-150-5p and miR-543-3p that target anti-inflammatory genes. J Immunol.

[159] Wang H, Wei Y, Yu D (2015). Control of lymphocyte homeostasis and effector function by the aryl hydrocarbon receptor. Int Immunopharmacol.

[160] Alvarez-Errico D, Vento-Tormo R, Sieweke M, Ballestar E (2015). Epigenetic control of myeloid cell differentiation, identity and function. Nat Rev Immunol.

[161] Mikami N, Kawakami R, Chen KY, Sugimoto A, Ohkura N, Sakaguchi S (2020). Epigenetic conversion of conventional T cells into regulatory T cells by CD28 signal deprivation. Proc Natl Acad Sci U S A.

[162] Yang N, Sen P (2018). The senescent cell epigenome Aging (Albany NY).

[163] Zhang W, Qu J, Liu GH, Belmonte JCI (2020). The ageing epigenome and its rejuvenation. Nat Rev Mol Cell Biol.

[164] Salminen A, Kaarniranta K, Hiltunen M, Kauppinen A (2014). Histone demethylase Jumonji D3 (JMJD3/KDM6B) at the nexus of epigenetic regulation of inflammation and the aging process. J Mol Med (Berl).

[165] Andriani GA, Almeida VP, Faggioli F, Mauro M, Tsai WL, Santambrogio L, Maslov A, Gadina M, Campisi J, Vijg J (2016). Whole chromosome instability induces senescence and promotes SASP. Sci Rep.

[166] Lyu G, Guan Y, Zhang C, Zong L, Sun L, Huang X, Huang L, Zhang L, Tian XL, Zhou Z (2018). TGF-β signaling alters H4K20me3 status via miR-29 and contributes to cellular senescence and cardiac aging. Nat Commun.

[167] Gaarenstroom T, Hill CS (2014). TGF-β signaling to chromatin: how Smads regulate transcription during self-renewal and differentiation. Semin Cell Dev Biol.

[168] Wang YQ, Li YM, Li X, Liu T, Liu XK, Zhang JQ, Guo JW, Guo LY, Qiao L (2013). Hypermethylation of TGF-β1 gene promoter in gastric cancer. World J Gastroenterol.

[169] Tian CQ, Chen L, Chen HD, Huan XJ, Hu JP, Shen JK, Xiong B, Wang YQ, Miao ZH (2019). Inhibition of the BET family reduces its new target gene IDO1 expression and the production of L-kynurenine. Cell Death Dis.

[170] Zhang H, Kuchroo V (2019) Epigenetic and transcriptional mechanisms for the regulation of IL-10. Semin Immunol 44:101324 10.1016/j.smim.2019.101324 PMC6981297 31676122

[171] Wajda A, Lapczuk-Romanska J, Paradowska-Gorycka A (2020). Epigenetic regulations of AhR in the aspect of immunomodulation. Int J Mol Sci.

[172] Huehn J, Beyer M (2015). Epigenetic and transcriptional control of Foxp3^+^ regulatory T cells. Semin Immunol.

[173] Zhao M, Qin J, Yin H, Tan Y, Liao W, Liu Q, Luo S, He M, Liang G, Shi Y (2016). Distinct epigenomes in CD4^+^ T cells of newborns, middle-ages and centenarians. Sci Rep.

[174] Sidler C, Woycicki R, Ilnytskyy Y, Metz G, Kovalchuk I, Kovalchuk O (2013). Immunosenescence is associated with altered gene expression and epigenetic regulation in primary and secondary immune organs. Front Genet.

[175] Goronzy JJ, Hu B, Kim C, Jadhav RR, Weyand CM (2018). Epigenetics of T cell aging. J Leukoc Biol.

[176] Horvath S, Raj K (2018). DNA methylation-based biomarkers and the epigenetic clock theory of ageing. Nat Rev Genet.

[177] Gross AM, Jaeger PA, Kreisberg JF, Licon K, Jepsen KL, Khosroheidari M, Morsey BM, Swindells S, Shen H, Ng CT (2016). Methylome-wide analysis of chronic HIV infection reveals five-year increase in biological age and epigenetic targeting of HLA. Mol Cell.

[178] Blagosklonny MV (2007). An anti-aging drug today: from senescence-promoting genes to anti-aging pill. Drug Discov Today.

[179] Salminen A, Kauppinen A, Kaarniranta K, Rahman I, Bagchi D (2014). Inflammaging signaling in health span and life span regulation: next generation targets for longevity. Inflammation, advancing age and nutrition.

[180] De Maeyer RPH, van de Merwe RC, Louie R, Bracken OV, Devine OP, Goldstein DR, Uddin M, Akbar AN, Gilroy DW (2020). Blocking elevated p38 MAPK restores efferocytosis and inflammatory resolution in the elderly. Nat Immunol.

[181] Sapey E, Greenwood H, Walton G, Mann E, Love A, Aaronson N, Insall RH, Stockley RA, Lord JM (2014). Phosphoinositide 3-kinase inhibition restores neutrophil accuracy in the elderly: toward targeted treatments for immunosenescence. Blood.

[182] Le Page A, Fortin C, Garneau H, Allard N, Tsvetkova K, Tan CT, Larbi A, Dupuis G, Fülop T (2014). Downregulation of inhibitory SRC homology 2 domain-containing phosphatase-1 (SHP-1) leads to recovery of T cell responses in elderly. Cell Commun Signal.

[183] Wang W, Wang L, Ruan L, Oh J, Dong X, Zhuge Q, Su DM (2018). Extracellular vesicles extracted from young donor serum attenuate inflammaging via partially rejuvenating aged T-cell immunotolerance. FASEB J.

[184] Aiello A, Farzaneh F, Candore G, Caruso C, Davinelli S, Gambino CM, Ligotti ME, Zareian N, Accardi G (2019). Immunosenescence and its hallmarks: how to oppose aging strategically? A review of potential options for therapeutic intervention. Front Immunol.

[185] Keren Z, Naor S, Nussbaum S, Golan K, Itkin T, Sasaki Y, Schmidt-Supprian M, Lapidot T, Melamed D (2011). B-cell depletion reactivates B lymphopoiesis in the BM and rejuvenates the B lineage in aging. Blood.

[186] Fahy GM, Brooke RT, Watson JP, Good Z, Vasanawala SS, Maecker H, Leipold MD, Lin DTS, Kobor MS, Horvath S (2019) Reversal of epigenetic aging and immunosenescent trends in humans. Aging Cell 18:e13028 10.1111/acel.13028 PMC6826138 31496122

[187] Draghiciu O, Lubbers J, Nijman HW, Daemen T (2015) Myeloid derived suppressor cells - an overview of combat strategies to increase immunotherapy efficacy. Oncoimmunology 4:e954829 10.4161/21624011.2014.954829 PMC4368153 25949858

[188] Li C, Jiang P, Wei S, Xu X, Wang J (2020). Regulatory T cells in tumor microenvironment: new mechanisms, potential therapeutic strategies and future prospects. Mol Cancer.

[189] Salminen A, Kaarniranta K, Kauppinen A (2018). Phytochemicals inhibit the immunosuppressive functions of myeloid-derived suppressor cells (MDSC): impact on cancer and age-related chronic inflammatory disorders. Int Immunopharmacol.

[190] Lee M, Park CS, Lee YR, Im SA, Song S, Lee CK (2014). Resiquimod, a TLR7/8 agonist, promotes differentiation of myeloid-derived suppressor cells into macrophages and dendritic cells. Arch Pharm Res.

[191] Azambuja JH, Ludwig N, Braganhol E, Whiteside TL (2019). Inhibition of the adenosinergic pathway in cancer rejuvenates innate and adaptive immunity. Int J Mol Sci.

[192] Kunisada Y, Eikawa S, Tomonobu N, Domae S, Uehara T, Hori S, Furusawa Y, Hase K, Sasaki A, Udono H (2017). Attenuation of CD4^+^CD25^+^ regulatory T cells in the tumor microenvironment by metformin, a type 2 diabetes drug. EBioMedicine.

[193] Qin G, Lian J, Huang L, Zhao Q, Liu S, Zhang Z, Chen X, Yue D, Li L, Li F et al (2018) Metformin blocks myeloid-derived suppressor cell accumulation through AMPK-DACH1-CXCL1 axis. Oncoimmunology 7:e1442167 10.1080/2162402X.2018.1442167 PMC5993496 29900050

